# The value of right ventricular pulmonary artery coupling in determining the prognosis of patients with sepsis

**DOI:** 10.1038/s41598-024-65738-2

**Published:** 2024-07-03

**Authors:** Qiang Ma, Caiyun Ding, Wei Wei, Chencheng Su, Bozheng Li, Zihao Zhou, Cui Chen, Biaohu Liu, Xia Zhang, Jingyi Wu

**Affiliations:** 1 Department of Ultrasound, First Affiliated Hospital of Wannan Medical College, No.2 Zheshan West Road, Wuhu, 241001 Anhui People’s Republic of China; 2 Department of Physiology, Wannan Medical College, Wuhu, People’s Republic of China; 3 Department of Neurology Intensive Care Unit, First Affiliated Hospital of Wannan Medical College, Wuhu, People’s Republic of China; 4 Department of Emergency Medicine, First Affiliated Hospital of Wannan Medical College, No.2 Zheshan West Road, Wuhu, 241001 Anhui People’s Republic of China

**Keywords:** Cardiopulmonary coupling, Hemodynamics, Echocardiography, Sepsis, Diagnostic markers, Heart failure

## Abstract

The outcomes of patients with sepsis are influenced by the contractile function of the right ventricle (RV), but the impact of cardiopulmonary interaction in ICU-mortality of sepsis patients remains unclear. This study aims to investigate the ICU-mortality impact of right ventricular-pulmonary artery (RV-PA) coupling in patients with sepsis. We employed echocardiography to assess patients with sepsis within the initial 24 h of their admission to the ICU. RV-PA coupling was evaluated using the tricuspid annular plane systolic excursion (TAPSE) to pulmonary artery systolic pressure (PASP) ratio. A total of 92 subjects were enrolled, with 55 survivors and 37 non-survivors. TAPSE/PASP ratio assessed mortality with an area under the curve (AUC) of 0.766 (95% CI 0.670–0.862) and the optimal cutoff value was 0.495 mm/mmHg. We constructed a nomogram depicting the TAPSE/PASP in conjunction with IL-6 and Lac for the joint prediction of sepsis prognosis, and demonstrated the highest predictive capability (AUC = 0.878, 95% CI 0.809–0.948). In conclusion, the TAPSE/PASP ratio demonstrated prognostic value for ICU mortality in sepsis patients. The nomogram, which combines the TAPSE/PASP, IL-6, and LAC, demonstrated enhanced predictive efficacy for the prognosis of sepsis patients.

## Introduction

Sepsis is a common critical illness in intensive care unit (ICU). According to The Third International Consensus for Sepsis and Septic Shock, it is defined as life-threatening organ dysfunction caused by a dysregulated host response to infection^1^. There are great differences in the number of deaths, age distribution and mortality rate of sepsis among different regions^2,3^. The incidence of sepsis in elderly individuals older than 65 years in Chinese intensive care units is as high as 57.5%, and the in-hospital mortality is as high as 30%^4^. Moreover, sepsis and septic shock represent significant and pressing global health challenges.

The definition of sepsis reflects its inherent heterogeneity. Heterogeneity is reflected in the different sources of infection in septic patients, the presence of complications and underlying diseases, and the early diagnosis and treatment^5,6^. Although it is difficult to accurately predict the prognosis of patients with sepsis, identifying important early indicators and adjusting treatment strategies are highly important for improving patient prognosis. Current studies have shown that many inflammation-based biomarkers, such as IL-6, IL-10, and HMGB1, can reflect the severity of sepsis and correlate well with clinical outcomes, but have proven to be lack generalizability to individual patients—in large part because these biomarkers have common characteristics of early inflammatory response but lack specificity^7^.

Ultrasound is playing an increasingly important role in the diagnosis and treatment of ICU patients^8,9^. Recent studies have shown that ultrasound indicators have certain value in evaluating the prognosis of patients with sepsis^10,11^. Patients with sepsis are prone to right ventricular dysfunction, which is more related to poor prognosis than left ventricular dysfunction^12,13^. Right ventricular-pulmonary artery (RV-PA) coupling is a measure of the functional match between right ventricular systolic function and afterload exerted by pulmonary vessels, and was assessed by the ratio of right ventricular end-systolic elasticity (Ees) to pulmonary artery elasticity (Es) measured by invasive pressure–volume loops^14^. However, this is difficult to achieve in routine clinical application. The ratio of tricuspid annular plane systolic excursion (TAPSE) to pulmonary artery systolic pressure (PASP) can be used to assess RV-PA coupling noninvasively and reliably^15,16^, and studies have shown that this parameter has important prognostic value for some diseases such as heart failure and severe pneumonia^17–19^. In our previous study, we found that patients with sepsis were prone to right heart dysfunction and pulmonary hypertension. Therefore, this study aimed to evaluate the cardiopulmonary interaction function of patients with sepsis in the intensive care unit by the TAPSE/PASP ratio, and to construct a nomogram by combining inflammatory indicators to predict the prognosis of septic patients.

## Methods

### Study population

This prospective observational study was conducted from September 2020 through September 2023 at a tertiary hospital ICU. All septic patients were screened for enrollment within 24 h after admission. Inclusion criteria are follows: (1) Patients with sepsis met the definition from the Third International Consensus Definitions for Sepsis and Septic Shock (Sepsis-3)3. Infection was confirmed through clinical and laboratory examinations. Additionally, a qSOFA score of ≥ 2 was required, which includes any two or more of the following three criteria: respiratory rate ≥ 22 breaths per minute; systolic blood pressure ≤ 100 mmHg; altered mental status (Glasgow Coma Scale score < 15). (2) age ≥ 18 years. Exclusion criteria included pregnancy, primary pulmonary arterial hypertension, liver cirrhosis, end-stage renal disease, abdominal hypertension and Patients who expired from causes other than septic shock, such as cardiovascular accidents or withdrawal of care by family members.

The study was approved by the ethics committee of our institution. Informed consent was obtained from family members. All methods employed in this study adhered to pertinent guidelines and regulations.

### Echocardiograph

Bedside echocardiography was performed for each patient on the first day of ICU admission, we considered the impact of positive pressure ventilation on right atrial load and right ventricle-pulmonary artery coupling ratio. Therefore, all ultrasound data were collected before the initiation of mechanical ventilation. All parameters were measured by an experienced physician (X.Zhang). The echocardiographic parameters included the right ventricular area change rate (FAC), and tricuspid annular plane systolic pressure excursion (TAPSE) (Supplementary Fig. 1A). The pulmonary artery systolic pressure (PASP) was calculated by the Eq. 4* (tricuspid regurgitation velocity squared) + right atrial pressure (RAP) (Supplementary Fig. 1B). We employed variations in the inferior vena cava inner diameter (IVC-D) to estimate right atrial pressure (RAP), which was estimated as 3 mmHg when IVC-D was < 21mm and the IVC respiratory variability (∆IVC) was < 50%.; When the IVC-D was ≥ 21mm and ∆IVC was ≥ 50%, the estimated valve was 15mmHg; If either criterion is met, the RAP is estimated as 8mmHg. The left ventricular ejection fraction (LVEF) was assessed using the Simpson biplane method. Each metric underwent a trifold measurement with subsequent calculation of the corresponding averages. A subset of patients, without knowledge of prior examination results, underwent a repeat assessment of right ventricle to pulmonary artery (RV-PA) coupling measurements by a second sonographer (W.Wei) to evaluate interobserver consistency.

### Other parameters collected

We systematically collected patient demographic information, encompassing variables such as heart rate (HR), blood pressure, respiratory rate. Simultaneously, Acute Physiology and Chronic Health Evaluation (APACHE) II and Sequential Organ Failure Assessment (SOFA) scores were calculated within the initial 24 h of hospital admission. Furthermore, we documented sepsis-related laboratory indicators, including red cell distribution width (RDW), blood lactate (LAC), interleukin-6 (IL-6), procalcitonin (PTC), and cardiac enzyme profiles.

### Statistical analysis

The data were analyzed with R 4.1.2 software. The quantitative data were tested for the Shapiro‒Wilk normality test, and normally distributed data are presented as the mean ± standard deviation. Between-group comparisons were conducted with the independent samples t-test. Nonnormally distributed data were expressed as medians (P25, P75), and the rank-sum test was used for comparisons between groups. The chi-square test was used for categorical variables. Multiple logistic regression was employed for factor selection, to assess independent contributors. The predictive efficacy of each independent factor was evaluated using receiver operating characteristic (ROC) curves, and Kaplan–Meier survival curves were plotted for key indicators. We constructed a nomogram for the joint diagnosis of key indicators and assessed the model's stability using a calibration curve. Delong's test was uesed to compare the diagnostic capabilities of various indicators. P < 0.05 indicated statistical significance.

### Ethics approval and consent to participate

This study was approved by the ethics committee of the Yijishan hospital, and we obtained informed consent from all patients or their family.

## Results

### Patient general characteristics

**Figure 1:**
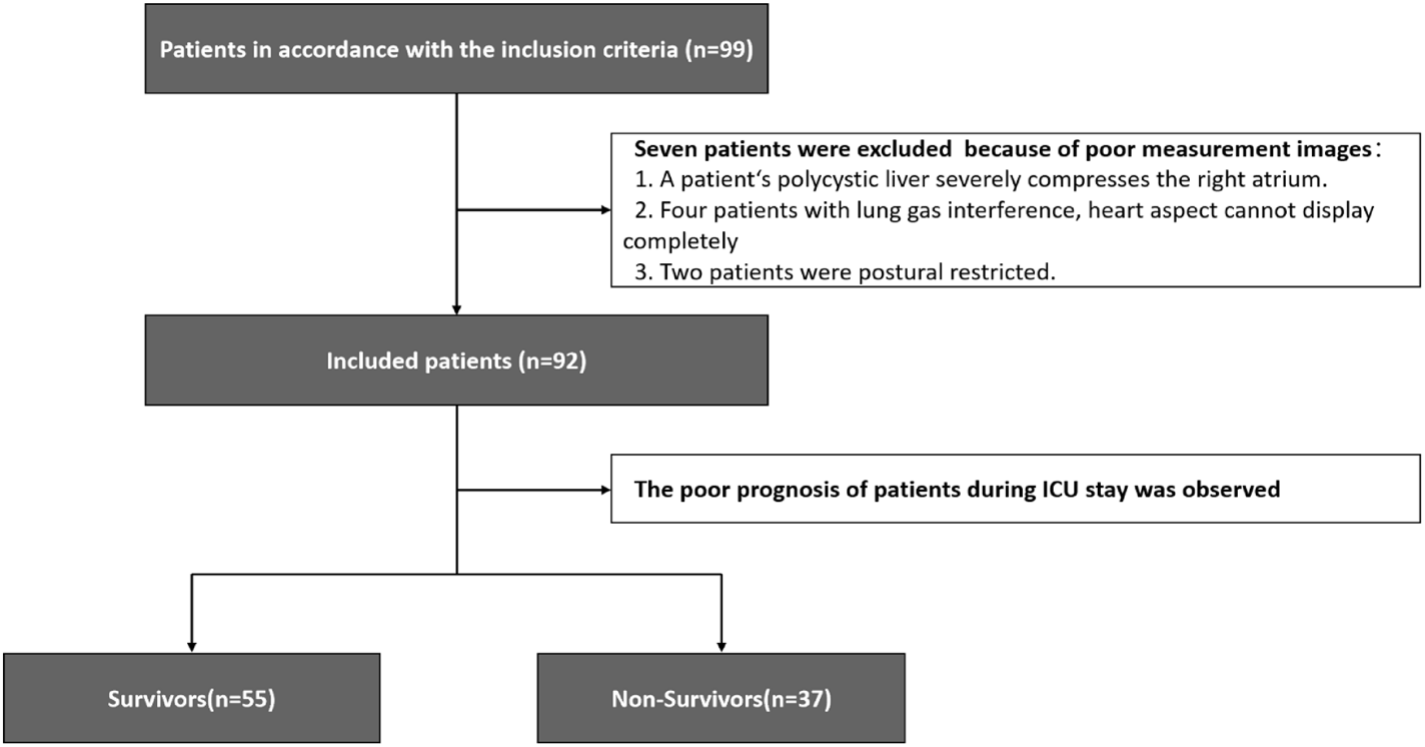
Flow chart.

From September 2020 to September 2023, 99 patients with sepsis were enrolled based on the inclusion criteria. Seven patients were excluded due to poor image quality, resulting in a final sample size of 92 patients, among whom 53 survived and 37 died during ICU admission (Fig. 1).

The characteristics of the population parameters were compared between the survival group and non-survivor group using the Student's t-test, Fisher’s exact test and the chi-square test. The results revealed statistically significant differences in the Apache II score (*p* < 0.001) and SOFA score (*p* < 0.001), while other indicators such as age, sex, education, blood pressure, and heart rate, were not significantly different. The most common source of infection was pulmonary, followed by gastrointestinal (Table 1).

**Table 1:** Characteristics of the Study Population.

	All patients (n = 92)	Survivors (n = 55)	Non- Survivors (n = 37)	*p*
Patients’ characteristics				
Age, year	68 (60, 75)	68 (61, 74.5)	68 (59, 75)	0.339
Female gender, n (%)	44 (48)	27 (49)	17 (46)	0.933^†^
SOFA	8 (5, 9.25)	7 (4, 9.5)	9 (7, 11)	< 0.001***
Apache II score	22 (16, 25)	20 (13.5, 23)	24 (21, 27)	< 0.001***
Length of stay at ICU, d	7 (4, 12.25)	9 (5, 13)	5 (3, 10)	0.019*
Mechanical ventilation, n (%)	72 (78)	39 (71)	33 (89)	0.145^†^
Source of infection, n				
Pulmonary	45	26	19	0.832^†^
Gastrointestinal	22	13	9	1^†^
Urinary	12	8	4	0.7561^†^
Biliary tract	7	4	3	1^†^
Acute pancreatitis	2	1	1	1^†^
Others	4	3	1	0.646^†^
Hemodynamic variables				
Heart rate, beats/min	101 (89.5, 112)	101 (90.5, 116.5)	102 (88, 112)	0.633
Respiratory rate	22 (19, 25)	22 (20, 25)	22 (19, 25)	0.649
Systolic blood pressure, mm Hg	94.1 ± 20.4	97.2 ± 18.8	89.6 ± 22.1	0.079
Diastolic blood pressure, mm Hg	55.6 ± 11.5	57.5 ± 10.8	53.3 ± 12.3	0.129

*Apache* acute physiology and chronic health evaluation; *SOFA* sequential organ failure assessment; *ICU* intensive care unit, Others including intracranial, mediastinum and skin infections.

^†^P value of Fisher-test.

### Echocardiographic and laboratory parameters

There were significant differences between the two groups in echocardiographic indices, including the TAPSE and TAPSE/PASP, as well as laboratory parameters such as LAC, and IL-6, with all p-values less than 0.001. Furthermore, FAC (*p* = 0.034), RVS (*p* = 0.023), PASP (*p* = 0.015), RDW (*p* = 0.004), and CK (*p* = 0.020) also exhibited statistically significant differences between the two groups (Table 2).

**Table 2:** Ultrasound and laboratory indexes.

	All patients (n = 92)	Survivors (n = 55)	Non- Survivors (n = 37)	*p*
Left ventricular function index				
LVEF, %	57.5 (51, 61.25)	58 (51, 62)	56 (48, 59)	0.126
LVS, cm/s	10.5 (8.8, 13.2)	10.9 (8.8, 14.05)	10.3 (8.6, 12.1)	0.198
E/e	10.5 (8.8, 12.22)	9.6 (8.5, 11.8)	11.3 (9.6, 12.5)	0.060
Right ventricular function index				
FAC, %	38 (33.75, 43.25)	39 (35.5, 45)	36 (33, 40)	0.034*
TAPSE, mm	14.5 ± 2.5	15.2 ± 2.5	13.3 ± 2.1	< 0.001***
RVS, cm/s	10.6 (9.5, 12.43)	11.1 (9.95, 13.1)	10.3 (9.2, 11.3)	0.023*
PASP, mmHg	28 (24, 34)	27 (23.5, 32.5)	32 (26, 36)	0.015*
TAPSE/PASP	0.50 (0.44, 0.62)	0.58 (0.47, 0.67)	0.45 (0.38, 0.48)	< 0.001***
Laboratory indexes				
RDW	14.55 (13.7, 16.38)	14 (13.45, 14.9)	15.3 (14.1, 18)	0.004**
LAC	2.85 (1.5, 4.53)	2 (1.4, 3.3)	3.9 (2.5, 5.6)	< 0.001***
IL-6	172 (54, 833)	100 (42, 222)	456 (197, 1517)	< 0.001***
NT-proBNP	708 (206, 1557)	815 (227, 2009)	431 (189, 1340)	0.450
Oxygen content	13.3 ± 4.2	13.8 ± 4.8	12.7 ± 4.1	0.223
CRP	126 (76, 182)	127 (73, 210)	122 (80, 169)	0.514
PCT	26.3 (3.4, 65.5)	14.2 (3.5, 66.3)	33.0 (3.3, 64.7)	0.802
CK	164 (63, 366)	234 (104, 451)	85 (58, 228)	0.020*
CK-MB	17.5 (9, 22.3)	18 (10.5, 45)	16 (7, 28)	0.353
LDH	228 (208, 478)	272 (200, 455)	309 (227, 543)	0.281
AST	42 (25, 85)	43 (23, 71)	39 (28, 195)	0.405

*LVS* Left Ventricular Strain, *RVS* Right Ventricular Strain, *NT-proBNP* N-Terminal pro B-type Natriuretic Peptide, *CRP* C-Reactive Protein, *PCT* Procalcitonin, *CK* Creatine Kinase, *CK-MB* Creatine Kinase-MB, *LDH* Lactate Dehydrogenase, *AST* Aspartate Aminotransferase.

*P value of T-test < 0.05.

^†^P value of Fisher-test.

### RV-PA coupling as a predictive factor

**Figure 2:**
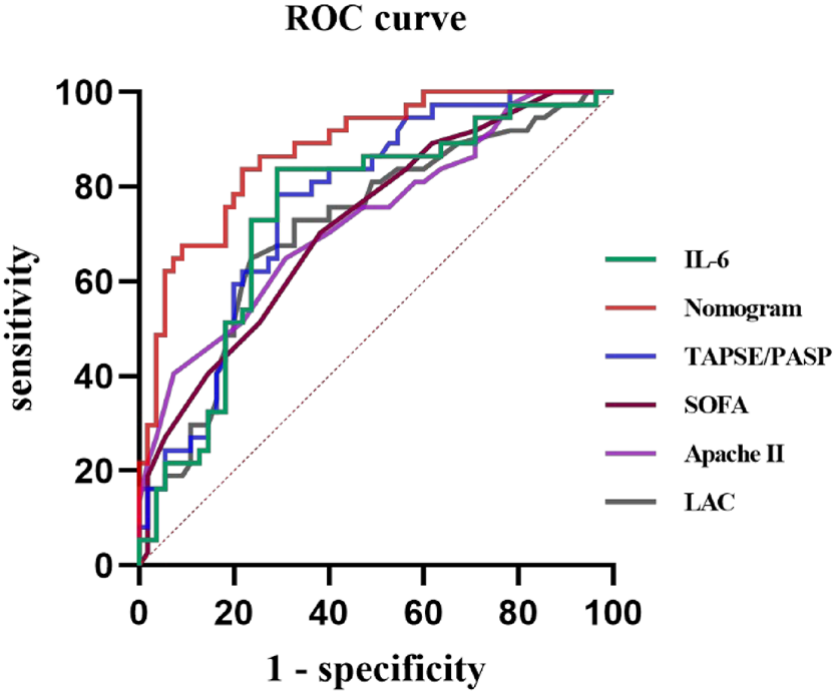
ROC curves of the nomogram, IL-6, TAPSE/PASP, SOFA, Apache II, LAC.

We plotted ROC curves for indicators with statistically significant differences, and the results demonstrated that the TAPSE/PASP had the highest AUC (0.952, CI 0.88–0.96) among the individual metrics (Fig. 2). The sensitivity, specificity, positive predictive value, and negative predictive value for the TAPSE/PASP were 0.83 (0.69–0.92), 0.64 (0.49–0.78), 0.71 (0.57–0.82), and 0.78 (0.62–0.90), respectively (Table 4). These results are consistent with previous studies, suggesting that the TAPSE/PASP is a reliable predictor of prognosis in patients with sepsis^20^. In addition, the Apache II score, SOFA score, as well as IL-6 and LAC also exhibited promising predictive performance, with AUCs of 0.729 (0.624–0.835), 0.721 (0.617, 0.825), 0.746 (0.641, 0.851), and 0.720 (0.612, 0.823), respectively. The optimal thresholds for Apache II scores, SOFA scores and TAPSE/PASP were 22.5. 7.5and 0.495, respectively (Table 3).

**Table 3:** The predictive value of models.

Variable	AUC %	Se %	Sp %	PPV %	NPV %	Best threshold
Apache II	72.9 (62.4, 83.5)	59 (42, 74)	75 (60, 86)	65 (47, 80)	69 (55, 81)	22.5
SOFA	72.1 (61.7, 82.5)	55 (40, 70)	76 (60, 87)	70 (53, 84)	62 (48, 75)	7.5
FAC	63.0 (51.4, 74.7)	30 (18, 44)	45 (29, 62)	43 (27, 61)	31 (19, 45)	36.5%
RDW	67.6 (56.1, 79.1)	59 (43, 74)	77 (63, 88)	70 (53, 84)	67 (53, 79)	14.7
LAC	72.0 (61.2, 82.3)	65 (47, 80)	76 (63, 87)	65 (47, 80)	76 (63, 87)	3.4
IL-6	74.6 (64.1, 85.1)	66 (51, 79)	87 (73, 95)	84 (68, 94)	71 (57, 82)	158
TAPSE/PASP	76.6 (67.0, 86.2)	83 (69, 92)	64 (49, 78)	71 (57, 82)	78 (62, 90)	49.5%
Nomogram	87.8 (80.9, 94.8)	72 (56, 85)	88 (75, 95)	84 (68, 94)	78 (65, 88)	0.379

**Figure 3:**
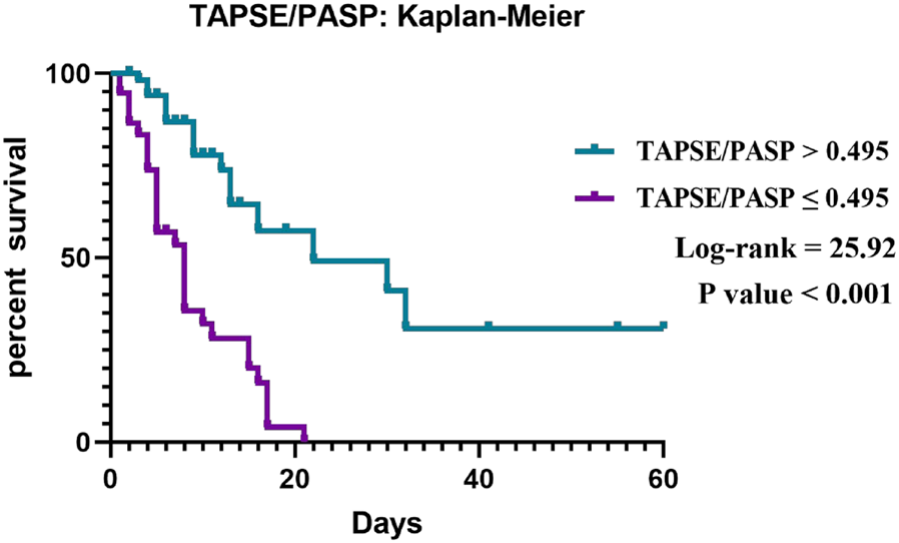
The Kaplan–Meier curves revealed a significantly higher ICU mortality rate in patients with TAPSE/PASP ≤ 0.495 mm/mmHg (log-rank: 25.92, *p* < 0.001).

We included indicators that demonstrated significant differences between the two groups in a multivariate logistic regression model. The results revealed that IL-6 (OR 1.00, 95% CI 1.00–1.00, *p* = 0.035), LAC (OR 1.38, 95% CI 1.07–1.89, *p* = 0.023), and TAPSE/PASP (OR 0.92, 95% CI 0.88–0.96, *p* = 0.002) were found to be independent prognostic factors for sepsis patients (Table 4). The Kaplan–Meier curves for estimated survival showed that ICU mortality was significantly greater in septic patients with TAPSE/ PASP ≤ 0.495 mm/mmHg than in patients with TAPSE/PASP > 0.495 mm/mmHg (HR: 25.92, *p* < 0.001) (Fig. 3).

**Table 4:** Results of multiple logistic regression.

Variable	Odds ratio	95% confidence interval	Z-value	*p*
Apache II	1.09	(0.95, 1.25)	1.233	0.218
SOFA	1.21	(0.92, 1.63)	1.326	0.185
FAC	0.96	(0.87, 1.06)	-0.708	0.480
RDW	1.09	(0.88, 1.37)	0.833	0.405
LAC	1.38	(1.07, 1.89)	2.271	0.023*
IL-6	1.00	(1.00, 1.00)	2.106	0.035*
TAPSE/PASP	0.92	(0.88, 0.96)	-3.129	0.002**

### Nomogram construction and effectiveness evaluation

**Figure 4:**
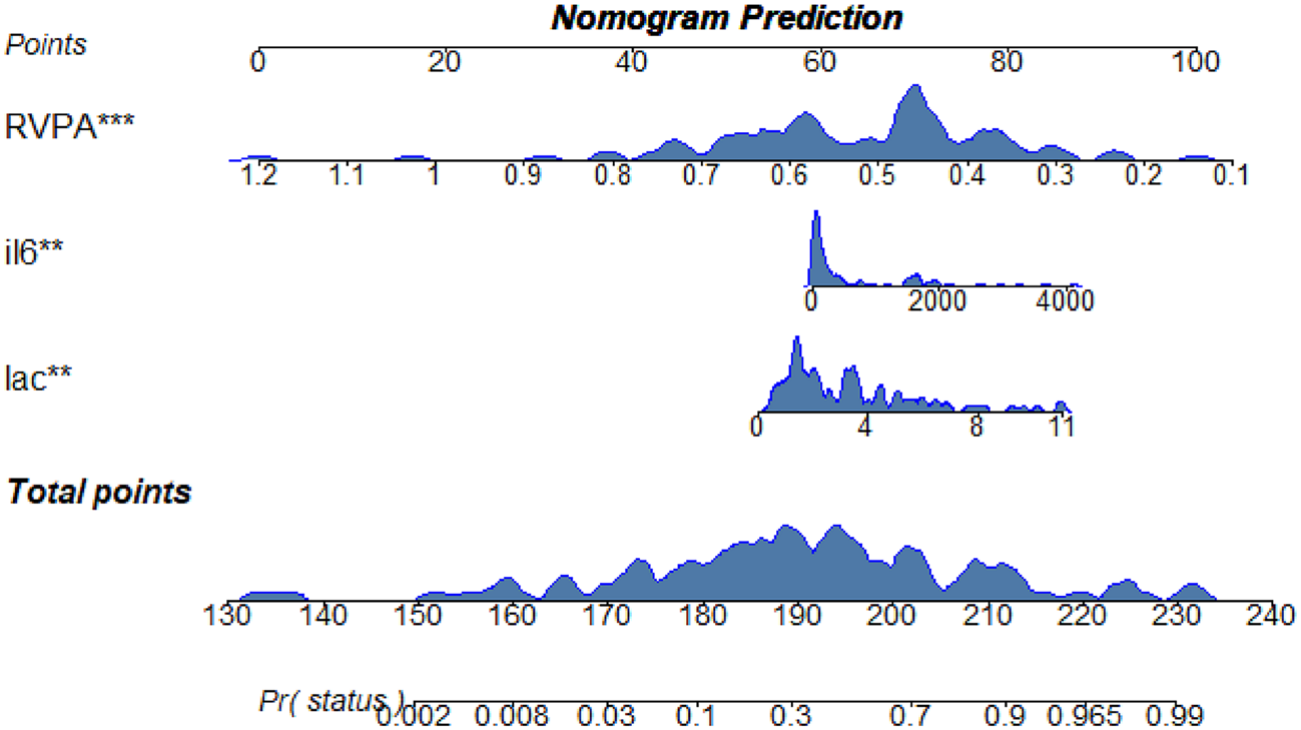
Nomogram established for predicting the ICU mortality of patients with sepsis.

We constructed a predictive model based on TAPSE/PASP, IL-6, and LAC, and generated a nomogram (Fig. 4). The ROC curve for the model showed an AUC of 0.878 (95% CI 0.809–0.948) (Fig. 2, Table 4). The Delong test revealed that the predictive performance was superior to each individual variable (*P* < 0.05). The calibration curve of the nomogram demonstrated high accuracy, with mean squared error (MSE) and mean absolute error (MAE) of 0.00061 and 0.02, respectively (Supplementary Fig. 2), indicating the stability of the model.

### Consistency test

In the present study, a cohort of 92 septic patients was analyzed, with a subset of 26 patients selected randomly for the purposes of repeatability and consistency analysis. Bland–Altman plots revealed the low mean biases (Supplementary Fig. 3). The results demonstrated good repeatability and consistency of the TAPSE/PASP measurements in patients with sepsis.

## Discussion

Hemodynamic disturbances are commonly encountered in critically ill patients with sepsis^21^. They play a pivotal role in the pathophysiology of sepsis, contributing to the complex cascade of events that may lead to multiple organ dysfunction syndrome (MODS) and increased mortality. Therefore, timely recognition and targeted management are crucial in addressing the challenges posed by hemodynamic instability in severe sepsis patients. Bedside ultrasound is playing an increasingly important role in the diagnosis and monitoring of septic patients^22^.

This study focused on right ventricular-pulmonary artery coupling (RV-PA) measured by echocardiography, combined with biochemical markers, to assess the early status of septic patients for predicting their prognosis. According to the general data analysis, nearly half of the septic patients had infections originating from the lungs, and a significant number of patients experienced concurrent right heart dysfunction. Additionally, the results indicated an increase in pulmonary artery systolic pressure in the non-survivor group (*p* = 0.015). The match relationship between myocardial contractility and ventricular afterload determines stroke volume, as well as the end-systolic pressure of the ventricle and arteries^23^. This interplay is a crucial determinant of cardiopulmonary function. RV-PA coupling is considered to reflect the matching between the right ventricle systolic function, represented by TAPSE, and its afterload, represented by PASP^24^. In the study, the TAPSE/PASP were significantly different between the survivor and non-survivor groups (*p* < 0.001). According to the subsequent multivariate logistic regression analysis, the TAPSE/PASP served as an independent predictor of the prognosis of septic patients, with an AUC of approximately 0.766 (0.670, 0.862) and an optimal cutoff value of approximately 0.495. These findings are consistent with the results of previous studies^20,25^. The Kaplan–Meier survival estimates revealed that ICU mortality was significantly greater in patients with TAPSE/PASP ≤ 0.495 mm/mmHg (log-rank 25.92, *p* < 0.001). In this study, the cause of death among the patients was septic shock. Septic shock is characterized by severe circulatory, cellular, and metabolic abnormalities, leading to systemic hemodynamic changes. These changes include reduced systemic vascular resistance and increased cardiac output, potentially resulting in right ventricular overload and dysfunction. This condition may be reflected in the multiple organ failure and elevated pulmonary artery pressures observed in the deceased patients. Different causes of death can affect echocardiographic parameters differently. For instance, patients who die from other causes such as acute myocardial infarction or pulmonary embolism might exhibit different patterns of ventricular dysfunction on echocardiography. However, in our cohort, the consistency of septic shock as the cause of death provides a clearer interpretation of the TAPSE/PASP ratio. It underscores the specific impact of septic shock on RV-PA coupling. Our findings support RV-PA coupling as a valuable prognostic tool in septic patients, particularly those at risk of developing septic shock. The TAPSE/PASP ratio reflects the dynamic interaction between right ventricular function and pulmonary artery pressure, serving as a reliable predictor of outcomes in this critically ill population.

Currently, there is substantial variation in the ability of various biomarkers and ultrasound indicators to predict the prognosis of septic patients across different studies, and the overall efficacy of these biomarkers tends to be moderate^26^. Comprehensive prediction and personalized treatment^27^ of multiple indicators of sepsis patients will be the future development trend^5^. Therefore, this study constructed a nomogram of TAPSE/PASP combined with biochemical markers IL-6 and LAC to diagnose the prognosis of sepsis patients. The results revealed that the predictive AUC of the nomogram was 0.878 (95% CI 0.809–0.948), surpassing the predictive efficacy of individual indicators, including SOFA score and Apache II score (DeLong test, *p* < 0.05). RV-PA uncoupling leads to the disturbance of cardiopulmonary interactions, exacerbates right ventricular dysfunction, decreases cardiac output and oxygen content, and may cause tissue hypoperfusion of small capillaries^28^. On the other hand, inflammatory factors in sepsis, such as IL-6 and lactate, act on blood vessels, triggering the formation of thrombi and constriction of small blood vessels in sepsis. On the other hand, septic inflammatory factors such as IL-6 and lac can act on blood vessels, which may cause serious complications such as disseminated intravascular coagulation (DIC), affecting tissue perfusion, and even leading to multiple organ failure and death in severe cases^29^. Therefore, sepsis, as a systemic immune response, is more accurate predicting the prognosis of sepsis patients through comprehensive evaluation of multiple indicators^30^.

This study has several limitations. First, this was a single-center study with a limited sample size. Further validation through larger sample sizes and continuous monitoring is essential. Second, this study chose to assess the condition of septic patients on the first day of ICU admission, before the initiation of mechanical ventilation. It is important to note that mechanical ventilation can significantly impact the RV-PA. During mechanical ventilation, the increase in inspiratory transpulmonary pressure raises RV afterload. When alveolar pressure surpasses left atrial pressure, pulmonary artery pressure becomes the downstream pressure opposing RV ejection. A study demonstrates a linear relationship between tidal volume (Vt), driving pressure, transpulmonary pressure, and the RV end-systolic pressure–volume relationship^31^. With increasing tidal volume, RV afterload also increases. Moreover, the increase in right heart afterload may affect the estimation of right atrial pressure by influencing right heart filling and pulmonary artery pressure. in subsequent research, we will delve into exploring the effects of mechanical ventilation and various mechanical ventilation parameters on the hemodynamics of patients with sepsis. Third, the presence of minimal or no tricuspid regurgitation in some patients may lead to partial bias in the measurement of PASP.

## Conclusions

The TAPSE/PASP exhibits substantial predictive value for the prognosis of patients with sepsis. Moreover, the nomogram constructed with TAPSE/PASP, IL-6, and LAC demonstrated enhanced predictive efficacy. These findings provide insights for enhancing the individualized prognostic assessment of patients with sepsis.

### Supplementary Information

Supplementary Information.

## Data Availability

The datasets used and/or analysed during the current study are available from the corresponding author on reasonable request.

## Abbreviations

ICU: Intensive care unit
LVEF: Left Ventricular Ejection Fraction
FAC: Fractional Area Change
TAPSE: Tricuspid Annular Plane Systolic Excursion
PASP: Pulmonary Artery Systolic Pressure
RV-PA: Right Ventricular Pulmonary Artery Coupling
RDW: Red Cell Distribution Width
LAC: Lactic Acid
IL-6: Interleukin-6
