# Supplementary material for: The value of right ventricular pulmonary artery coupling in determining the prognosis of patients with sepsis
Source: Sci Rep. 2024 Jul 3;14:15283. doi: 10.1038/s41598-024-65738-2 (PMC11222489; doi:10.1038/s41598-024-65738-2)
Supplement: Supplementary file 1 — Supplementary Information. [file 41598_2024_65738_MOESM1_ESM.docx]

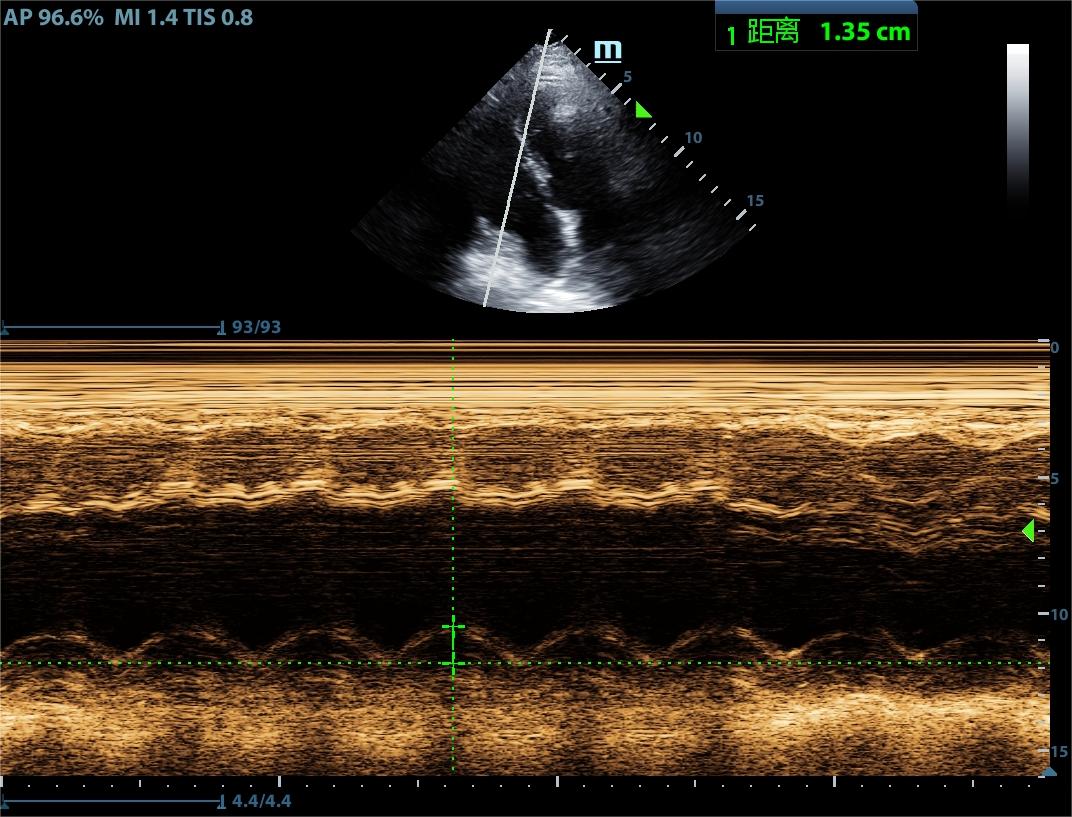


Supplementary Figure 1: TAPSE


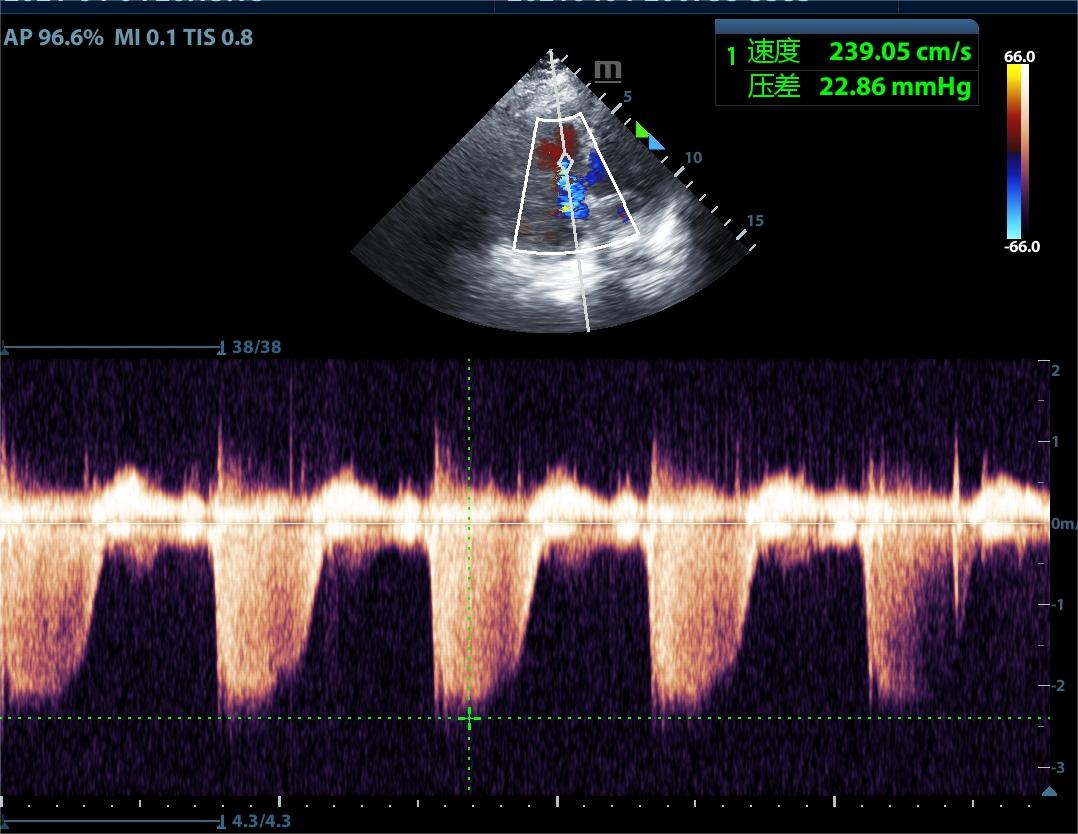


Supplementary Figure 2: PASP


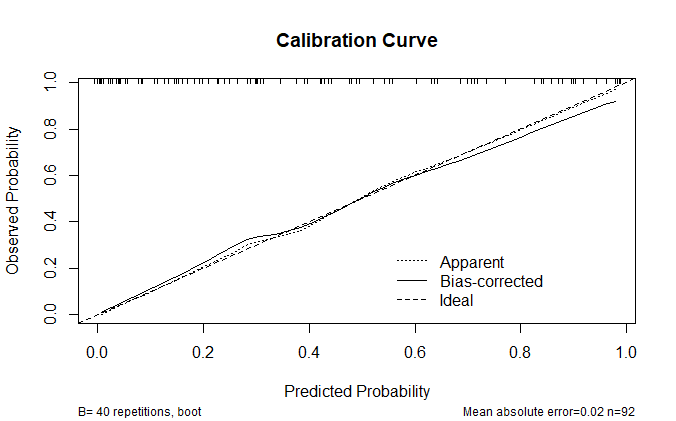


Supplementary Figure 3: Calibration Curve


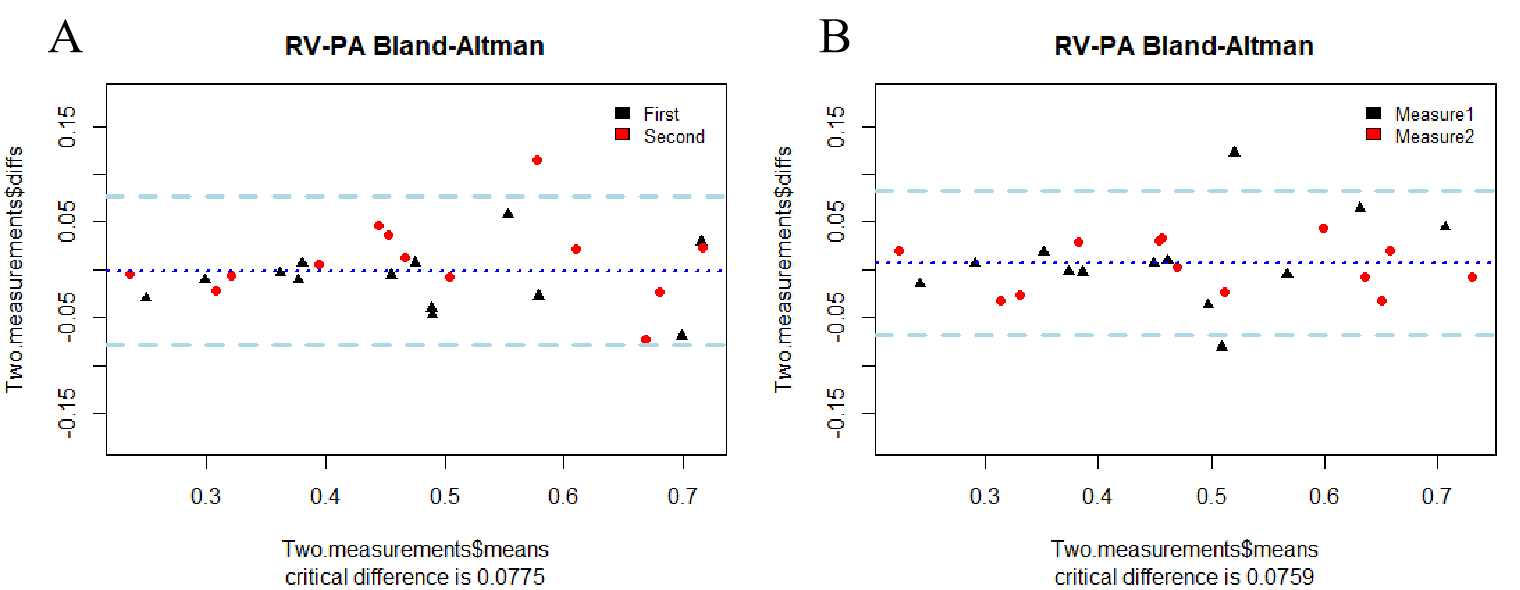


Supplementary Figure 4: Bland-Altman
